# Improved quality of management of eclampsia patients through criteria based audit at Muhimbili National Hospital, Dar es Salaam, Tanzania. Bridging the quality gap

**DOI:** 10.1186/1471-2393-12-134

**Published:** 2012-11-21

**Authors:** Hussein Lesio Kidanto, Peter Wangwe, Charles D Kilewo, Lennarth Nystrom, Gunnila Lindmark

**Affiliations:** 1 Department of Obstetrics and Gynaecology, Muhimbili University of Health and Allied Sciences, Dar es Salaam, Tanzania; 2 Department of Obstetrics and Gynaecology, Muhimbili National Hospital, Dar es Salaam, Tanzania; 3 Division of Epidemiology and Global Health, Department of Public Health and Clinical Medicine, Umeå University, Umeå, Sweden; 4 Department of Women’s and Children’s Health, Academic Hospital, Uppsala, Sweden

**Keywords:** Eclampsia, Pre-eclampsia, Criterion-based audit, Quality of care, Tanzania

## Abstract

**Background:**

Criteria-based audits (CBA) have been used to improve clinical management in developed countries, but have only recently been introduced in the developing world. This study discusses the use of a CBA to improve quality of care among eclampsia patients admitted at a University teaching hospital in Dar es Salaam Tanzania.

**Objective:**

The prevalence of eclampsia in MNH is high (≈6%) with the majority of cases arriving after start of convulsions. In 2004–2005 the case-fatality rate in eclampsia was 5.1% of all pregnant women admitted for delivery (MNH obstetric data base). A criteria-based audit (CBA) was used to evaluate the quality of care for eclamptic mothers admitted at Muhimbili National Hospital (MNH), Dar es Salaam, Tanzania after implementation of recommendations of a previous audit.

**Methods:**

A CBA of eclampsia cases was conducted at MNH. Management practices were evaluated using evidence-based criteria for appropriate care. The Ministry of Health (MOH) guidelines, local management guidelines, the WHO manual supplemented by the WHO Reproductive Health Library, standard textbooks, the Cochrane database and reviews in peer reviewed journals were adopted. At the initial audit in 2006, 389 case notes were assessed and compared with the standards, gaps were identified, recommendations made followed by implementation. A re-audit of 88 cases was conducted in 2009 and compared with the initial audit.

**Results:**

There was significant improvement in quality of patient management and outcome between the initial and re-audit: Review of management plan by senior staff (76% vs. 99%; P=0.001), urine for albumin test (61% vs. 99%; P=0.001), proper use of partogram to monitor labour (75% vs. 95%; P=0.003), treatment with steroids for lung maturity (2.0% vs. 24%; P=0.001), Caesarean section within 2 hours of decision (33% vs. 61%; P=0.005), full blood count (28% vs. 93%; P=0.001), serum urea and creatinine (44% vs. 86%; P=0.001), liver enzymes (4.0% vs. 86%; P=0.001), and specialist review within 2 hours of admission (25% vs. 39%; P=0.018). However, there was no significant change in terms of delivery within 24 hours of admission (69% vs. 63%; P=0.33). There was significant reduction of maternal deaths (7.7% vs. 0%; P=0.001).

**Conclusion:**

CBA is applicable in low resource setting and can help to improve quality of care in obstetrics including management of pre-eclampsia and eclampsia.

## Background

About 50,000 maternal deaths occur yearly due to eclampsia, the majority in countries with limited resources and low standard of health care [1,2]. Maternal mortality rates and quality of health care in low- and middle-income countries is different, however, we both talk of achieving Millennium development goal (MDG) despite having different levels of resources. Several strategies have been identified for reducing maternal and perinatal mortality e.g. through high quality antenatal care, delivery attended by trained health personnel and provision of both basic and comprehensive emergency obstetrical care. These strategies are the same both in poor and good resource countries but the difference is the quality of service provided due to limited resources in developing countries [3].

Improvement of clinical management using locally available resources for the management of pre-eclampsia and eclampsia have been proved to reduce perinatal and maternal morbidity and mortality [4]. A criteria-based audit (CBA) has been used for many years to improve clinical management in developed countries, but has just recently been introduced in developing countries. Recent CBAs in Uganda and Malawi of management of pre-eclampsia and emergency obstetrics demonstrated that it is feasible to improve the quality of obstetric care by using locally available resources [5,6].

The prevalence of eclampsia in MNH is high (≈6%) with the majority of cases arriving after start of convulsions. A special unit in close proximity to the delivery ward takes care of the eclamptic cases. In 2004–2005 the case-fatality rate in eclampsia was 5.1% of all pregnant women admitted for delivery (MNH obstetric data base). Therefore, a quality improvement process of the eclampsia management and care was initiated using a CBA [7,8].

The aim of this study was to evaluate the improvements of the management of eclampsia patients after implementation of recommendations made based on the initial CBA by a re-audit at a tertiary teaching hospital in Dar es Salaam, Tanzania.

## Material and methods

### Study design, study period and study population

The initial CBA was performed April-December 2006. After implementing the recommended changes the re-audit CBA was perfomed between January-March 2009 using case notes of women that meet inclusion criteria, that is, all cases with eclampsia that were admitted to the labour ward at Muhimbili National Hospital (MNH) during the study period.

### Setting

Muhimbili National Hospital is a teaching hospital for Muhimbili University of Health and Allied Sciences, and one of four large consultant hospitals in the United Republic of Tanzania. It is situated in Dar es Salaam, which according to the 2002 census had a population of about 2.5 million and an annual population growth rate of 4.3% [9]. The hospital serves as a referral centre for the city of Dar es Salaam and the neighboring coastal region. The annual number of deliveries in the hospital is about 10,000, (≈30/day), out of which 80% are low-risk deliveries. Every month there is a perinatal mortality meeting involving all members of the department of obstetrics and representatives from the neonatal ward, where the perinatal mortality trend as well as selected perinatal mortality cases are presented and discussed.

There are three shifts for nurses working in the labour ward, each with six midwives. One specialist obstetrician, one consultant obstetrician and two residents (house officer**s**) are on call every day. After a normal vaginal delivery the mothers and babies are observed in hospital for 6–10 hours. During this time the babies also get BCG and polio vaccinations before being discharged. Babies delivered by caesarean section (CS) or those with low Apgar score (<7**)** are admitted to the neonatal ward, which is just one floor up from the labour ward. The labour ward also admits sick babies from other hospitals.

## Method

### Audit procedure

**Figure 1:**
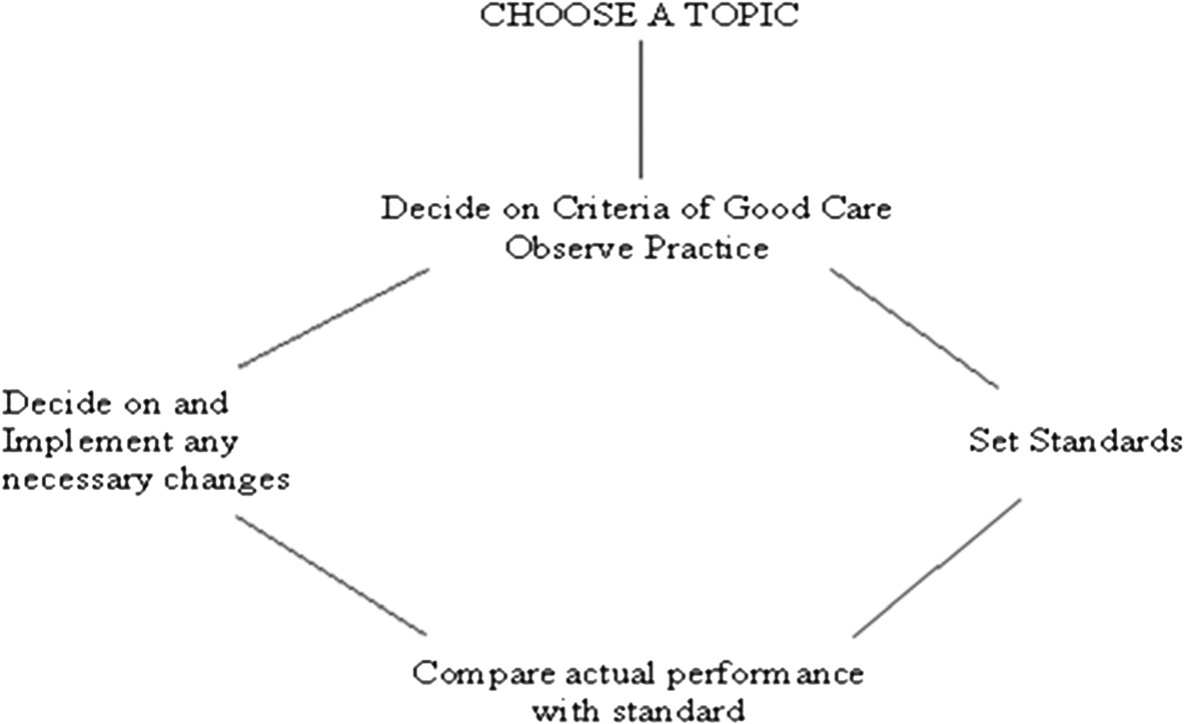
Audit cycle.

The audit was performed following a criteria-based audit framework [10]. Clinical audit is the systematic and critical analysis of the quality of medical care, including the procedures used for diagnosis and treatment, the use of resources and the resulting outcome and quality of life for the patient. It follows this cycle of events: Figure 1.

#### Setting the standard (step 1)

In March 2006 a meeting was convened in the department with all doctors and nurses/midwives to agree on evidence-based criteria on management of eclampsia using the Ministry of Health (MOH) guidelines [11], local management guidelines, the WHO manual [12] supplemented by the WHO Reproductive Health Library CD-ROM no.8, standard textbooks, the Cochrane database and reviews in peer reviewed journals. A set of 14 standards (Table 1) was developed which addressed quality issues related to the management of eclampsia. The standards were set according to the prevailing local setting and according to available resources. Two workshops were conducted to inform the department members on the standards. Each workshop involved 50 doctors, nurses, and midwives.

**Table 1:** Summary of criteria for optimal management of eclampsia and severe pre eclampsia at the labour ward, Muhimbili National Hospital

1	The patient should be seen (by a resident/registrar) within 1 hour of arrival in eclampsia ward and thorough history documented including age, parity, gestational age, number of fits, time of first fit, source of admission, current pregnancy history and past medical history.
2	General clinical state (pulse, blood pressure, temperature etc.) on admission should be recorded by a senior admitting nurse including documentation of treatments received or came with and time it started and any treatment given as emergency before doctor’s order
3	A specialist or consultant obstetrician should be involved in planning the management by reviewing the resident’s plan within 1 hour
4	Anti-hypertensive treatment should be given to all patients with severe hypertension (diastolic blood pressure (BP) ≥110mmHg)
5	The treatment and prophylaxis of seizures should start immediately with magnesium sulphate and continue for 24 hours after last fit or delivery depending on which comes first (Dose as per eclampsia treatment protocol)
6	Respiratory rate and tendon reflexes should be monitored every half an hour when magnesium sulphate is used
7	Ante/intrapartum fluid balance chart should be maintained and input output recorded
8	Full blood count, renal, and liver function tests as well as urinalysis should be done at least once (Full blood picture, urine for albumin test, serum creatinine, urea, liver enzymes (Alanine Aminotransferase (ALT), Aspartate Aminotransferase (AST) and Alkaline phosphatise))
9	The foetal heart rate should be monitored every 30 minutes in all undelivered patients
10	Steroid therapy should be given in all pregnancies where gestational age is 28–34 completed weeks in case of a need for prolongation of pregnancy
11	The patient should be delivered within 12h of the first convulsion
12	Monitoring BP and urine output should continue for at least 48 hours after delivery

#### Step no 2

The aim of this step was to evaluate the current practice. Data were collected by a medical doctor (senior resident) trained for the purpose. Socio-demographic data were collected as documented in case notes. Case files were reviewed and records compared against the standards, comprehensive information like antenatal care, events during antepartum and intrapartum period as well as pregnancy outcome was obtained by the principal investigator from case notes and summary. Furthermore, information was collected on gestational age at delivery, number of eclamptic seizures, blood pressure on admission, proteinuria at the antenatal clinic and on admission, gestational age at diagnosis of eclampsia, use of antihypertensive drugs, delivery complications, mode of delivery, Apgar score, birth weight, time interval between admission and delivery, and perinatal and maternal morbidity and mortality. Records on all babies referred to the neonatal ward were reviewed to collect data on early neonatal deaths. Cause of perinatal death was selected from the case notes as recorded by the doctor who certified the death. A post-mortem examination was not performed. The admission book was used to check if all admitted cases were included.

#### Analysis of the initial audit and development of recommendations (Step no 3)

After analysis of data from the initial audit the results were presented to members of the department at a specially convened department meeting held in September, 2007.The gaps between current practice and standards were discussed and recommendations for improvements were made (Table 2). The implementation of the recommendations commenced in December, 2007 and was run for ten months. This involved assigning a specialist to manage cases in the ward with assistance of an intern and resident doctors. This improved the consultation and decision making process between cadres in the managing team (doctors, midwifes). Midwives were trained to start the management of patients as soon as they were admitted by initiating the monitoring of vital signs, insertion of Foley catheters, setting IV lines and initiation of medications to control high blood pressure and fits. A register was developed to trace patients scheduled for caesarean section so that unnecessary delays were avoided. Laboratory tests were collected and results traced, consumables e.g. urine sticks for measuring protein were made available.

**Table 2:** Changes made according to recommendations made in the previous audit

1.	An obstetrician, a resident and an intern were assigned to cover the eclampsia ward for 24 hours
2.	Urine dipsticks were purchased and made available in the ward
3.	A protocol for management of eclampsia and severe pre-eclampsia as well as steroid use was displayed in the ward
4.	Because records were often poor, commitment to proper recording and use of the partogram was emphasized
5.	Decision to surgery time to be shortened and a tracer method (a register) was designed to note the time of decision, time of transfer to theater and actual time of surgery to detect point of delay.
6.	Management of eclampsia or severe pre-eclampsia was emphasized to be started immediately on arrival and the treatment plan to be reviewed by specialist within two hours
7.	Laboratory tests (Full blood count, liver enzymes, urinalysis, and kidney function tests) were made routine for every patient admitted to the eclampsia ward

#### The re-audit (Step 4)

After implementation of the recommendations from the initial audit, a re-audit was conducted from January, 2009 to March, 2009 to assess the progress. The data collection procedure was done in the same way as in the initial audit [13].

### Main outcome measures

Main outcome measures were Apgar score < 7 or >/= to 7 at 5th minute, time interval between admission and delivery, time interval between decision and performance of CS for those delivering by CS, time from admission to assessment by specialist and perinatal and maternal morbidity and mortality.

### Statistical methods

Data were extracted using a data extraction sheet containing all variables of interest and entered into a data based created in SPSS software. Analysis was performed using PASW statistics version 18. Differences in maternal characteristics between women participating in the initial audit and the re-audit were analysed using chi-square test and differences in the percentage of women that attained the recommended standard at the initial audit and the re-audit were analysed using Student’s t-test. Level of statistically significance was set at p<0.05.

### Ethical clearance and ethical issues

Procedures for the study has closely followed approved ethical guidelines for biomedical research involving human subjects’(including human material or human data) in compliance with the Helsinki Declaration ( http://www.wma.net/en/30publications/10policies/b3/index.html). All reviewed cases notes were coded and anonymized; Confidentiality was guaranteed by not using names of the clients in the analysis. Ethical clearance for the study was granted by Muhimbili University of Health and Allied Sciences research and ethical committee and permission to conduct the study was from Muhimbili National Hospital administration.

### Definitions

1. Early neonatal death =Death <7 days of life

2. Birth asphyxia =Apgar score <7 at 5 minutes after birth with cardio respiratory and neurological depression

3. HELLP syndrome =A clinical entity of preeclampsia associated with Haemolysis, Elevated Liver Enzymes and Low Platelets

4. Eclampsia =. Convulsion >24 weeks of pregnancy with hypertension (BP>140/90mmHg) and proteinuria

5. Preterm delivery =Delivery <37 completed weeks of pregnancy

6. Gestational age at delivery=Calculated according to Naegele’s formula as the duration of the pregnancy in weeks, i.e. from the first day of the last normal menstrual period to the date of delivery. Ultrasound records were also used for those who had a first or early second trimester scan.

7. Proteinuria = Urinary excretion of 0.3 g of protein or greater in a 24-hours urine specimen or +2 or greater on urine dip specimen.

## Results

We analysed 389 cases of eclampsia in the initial audit and 88 cases in the re-audit (Table 3). The re-audit showed significant improvements in many of the set criteria for quality care of eclampsia (Table 4) as compared to the initial audit. Specialist review within 2 hours of admission increased from 25% in the initial audit to 39% (p=0.018) in the re-audit and percentage of women whose operative delivery was planned and achieved within 2 hours increased from 33% (26/78) to 61% ( 23/38) (p=0.005).

**Table 3:** Maternal characteristics of women admitted to the eclampsia ward in the initial audit and re-audit

	**Initial audit**		**Re-audit**		**p- value**
	**No**	**%**	**No**	**%**	
*Age (years):*					
15-24	262	67	20	23	<0.001
25-34	94	24	54	61	
≥35	33	8.5	14	16	
*Parity:*					
0	260	67	59	67.0	<0.001
1-2	106	27	22	25	
≥3	23	5.9	7	8.0	
*Gestational age (weeks):*					
24-32	55	14	21	24	0.024
33-36	154	40	17	19	
≥37	178	46	50	57	
Unknown	2	0.5			
*No of ANC visits:*					
0	31	7.9	7	7.9	0.013
1-2	152	39	22	25	
≥3	206	53	59	67	
*Mode of delivery:*					
SVD	278	72	47	53	0.001
CS	76	20	38	43	
ABD	8	2	2	2.3	
others	27	6.0	1	1.1	
*Maternal outcome*					
Dead	30	7.7	0	0	0.001
Alive	359	92.3	88	100	

*SVD=Spontaneous vertex deliver, CS=Caesarean section, ABD=assisted breech delivery and ANC= Antenatal clinic, P-value for chi-square test of association.

**Table 4:** Number and percent women at the initial audit (n=389) and re-audit (n=88) that attained the standard

**Standard**	**Initial audit**		**Re-audit**		**p-value**
	**No**	**%**	**No**	**%**	
Detailed history and documentation	381	98	87	99	0.57
Management plan by senior staff	297	76	87	99	<0.001
Use of MgSO_4_	389	100	88	100	1
Initiating drug treatment in severe hypertension	243/245	99	88/88	100	0.40
Specialist review within 2 hours of admission	99	25	34	39	0.018
BP monitored	389	100	88	100	1
Urine for albumin test	236	61	87	99	<0.001
Fluid balance chart should be maintained for 48 hours	385	99	88	100	1
Respiration rate monitored	389	100	88	100	1
Treatment with steroids for lung maturity	3/132	2.0	5/21	24	<0.001
CS within 2 hours of decision	26/78	33	23/38	61	0.005
Full blood count to all admitted patients	108	28	82	93	<0.001
Serum urea and creatinine to all patients	170	44	76	86	<0.001
Liver function test to all patients	16	4	76	86	<0.001
Delivery within 24 hours of admission	*235/343	69	*47/75	63	0.40
Deep tendon reflex assessment	2	0.5	6	6.8	<0.001
Proper use of partogram	*257/343	75	*68/75	91	0.003

*number observed per delivery.

P-value for Student’s t-test of difference in prevalence between the audits.

The use of partogram for monitoring labour improved from 75% (257/343) to 91% (68/75) (p=0.003) from the initial audit to the re-audit respectively. Percentage of women with full blood count performed increased from 28% in the initial audit to 93% in the re-audit (p<0.001), whereas measurement of serum creatinine and urea and liver enzymes improved from 44% to 86% and from 4.1% to 86%, respectively (p<0.001).

The standard for the treatment of eclampsia (MgSO_4_) and monitoring of blood pressure and respiratory rate in eclamptic patient was maintained at 100% in the initial and in the re-audit.

There were 30 maternal deaths from eclampsia in the initial audit that occurred during antepartum (n=7), intrapartum (n=14) and postpartum (n=9) and no maternal death occurred during the re-audit (p=<0.001). Out of 161 perinatal deaths recorded in the initial audit, 37% were fetal deaths before admission (macerated stillbirths), 16% were fetal deaths that occurred after admission or during labour (fresh stillbirths), 29% were early neonatal deaths, and 18% were unclassified due to poor documentation. In the re-audit out of 32 perinatal deaths 11 (34%) were macerated stillbirths, 9 (28%) were fresh stillbirths, and 12 (38%) were early neonatal deaths.

## Discussion

There was a significant improvement in quality of patient management and outcome in the re-audit within the limited resources available. These improvements were observed within one year following implementation of the recommendations from the initial audit. The improvements were seen for all aspects of care; in particular for intrapartum monitoring where partographs were used and planned delivery was more common. The most important change in outcome was the improved survival of the mothers, there was no maternal death in the re audit.

Efforts to improve maternal and newborn quality of care which are self driven without mobilizing external resources are necessary in sub-Saharan countries where maternal mortality and morbidity is still unacceptably high. Reorganization of available human resources (physicians and nurses) in all levels accompanied by self motivation played a key role in the achievement in this study. A CBA was used for problem identification, resource mobilization and problem solving by prioritizing. The method allows the service providers themselves to conduct quality assessment aiming at achieving targeted goals which are locally acceptable and using their own local resources [7,8]. Self assessment of an institution before and after auditing is important because this help one to discover unutilized available resources by re-organization before calling for external funding [11,12].

In most obstetric complications, especially severe ones like eclampsia, quick recovery depends on the proper initial action. Apart from delivery, correct drug dosages, IV Fluid type and volume administered being critical to outcome, improvement in labour monitoring using the partogram is also pivot in the management of all patients who were undelivered hence the general improvement in the re-audit [14-16]. Management of eclamptic patient must focus on three components namely: monitoring maternal clinical status and fetal wellbeing by using a simple and locally accepted tool (partograph), performing laboratory tests for vital maternal functions and providing appropriate obstetrical intervention [13,17].

Because of the multiple organ injuries caused by the pathophysiology of eclampsia adequate laboratory investigation is important for patient management. The use of laboratory tests increased after the initial audit. The improvements were similar to those found in a study in Uganda [5]. Despite the constraints described by Ronsmans [18] for successful implementation of clinical audit in the low resource countries, this study has proven otherwise without additional external resources. Re-organization of the daily routines and setting of priorities were pivotal to success. Thus an obstetrician, a resident and an intern doctor were assigned to cover the eclampsia ward. This improved the quality of management of the eclampsia patient as evidenced by good maternal outcome. Although compliance to the new guideline can be difficult for rotating junior medical staff (interns and residents) [10] the assigned obstetrician ensured that there was a uniform care for all patients admitted to the eclamptic ward by following the recommended guideline. This made implementation possible. In particular the availability of doctors contributed to the improvement of skilled assessment, decision on interventions and reduction of waiting time for CS.

The fact that documentation and details of case note taking increased to 99% lack of significance is due to the limited number of cases in second audit. Compared to the previous audit achievement of delivery within 24 hours was 69% vs. 63%. Our findings are in line with CBA by Weeks et al. in Kampala [5]. They found that, despite the general improvement in the re-audit, documentation was still poor, but did not affect the whole process of auditing. In our study out of the 14 criteria 12 were attained giving an average performance score of about 86%.

Although the findings from this study are encouraging, the main limitations included fewer cases in re-audit than in the initial audit; this was the result of new eclampsia units opened in the three municipal hospitals that are the main referring hospital.

Quality improvement can be expensive, but this audit proved that using available human resources with involvement of all relevant staff in the hospital can achieve a very satisfactory result without additional expense.

## Conclusion

CBA is applicable in a low resource setting and can help to improve quality of care in obstetrics including management of pre-eclampsia and eclampsia. The process and solutions to the identified problems are inexpensive and attainable within a limited time. Reorganization of resources and priorities in problems solving is the key to standardized quality of care.

## Competing interests

The authors declare that they have no competing interests.

## Authors’ contributions

HLK, conceived the study, and participated in its design and coordination and helped to draft the manuscript. PW, Carried out the data collection and involved in the analysis and drafted the first manuscript. CDK, participated in data analysis and final manuscript development. LN participated in the analysis of data, and development of the manuscript. GL reviewed the final manuscript. All authors read and approved the final manuscript.

## Pre-publication history

The pre-publication history for this paper can be accessed here:

http://www.biomedcentral.com/1471-2393/12/134/prepub
